# Pharmacokinetic Evaluation of a Novel Donepezil-Loaded Dissolving Microneedle Patch in Rats

**DOI:** 10.3390/pharmaceutics14010005

**Published:** 2021-12-21

**Authors:** Naveed Ur Rehman, Chanwoo Song, Junhyeong Kim, Inhwan Noh, Yun-Seok Rhee, Hye Jin Chung

**Affiliations:** College of Pharmacy and Research Institute of Pharmaceutical Sciences, Gyeongsang National University, Jinju 52828, Korea; naveed.rehman50@gmail.com (N.U.R.); cwsong@gnu.ac.kr (C.S.); jhk6914@naver.com (J.K.); oinhoan323@gnu.ac.kr (I.N.)

**Keywords:** donepezil, dissolving microneedles, Alzheimer’s disease, sustained release

## Abstract

Research on the development of dissolving microneedles (DMNs) has focused on bolus drug delivery, with little attention on sustained release. Here, we evaluated the sustained release, absorption pattern, and effective drug permeation of a novel donepezil-loaded DMN patch through an in vivo investigation on rats. The applications of DMN patches to the shaved skin of rats for 1 week and 1 h were compared with oral donepezil administration to assess their sustained release capabilities. We used a validated liquid chromatography–tandem mass spectrometry method to quantify donepezil in the plasma. We found that the microneedle arrays effectively delivered donepezil across the skin, with dissolution observed within 1 h of application. Furthermore, skin irritation test showed that the patches produced no irritation response. The DMN arrays also effectively increased drug permeation and demonstrated sustained release and absorption of donepezil from DMN patches. These patches allow extended dosing intervals, reduced gastrointestinal adverse effects, and convenient self-administration to mitigate poor drug compliance, making them beneficial for the treatment of elderly patients with Alzheimer’s disease.

## 1. Introduction

The field of microneedles is an emerging research area that combines the advantages of transdermal patches and the effectiveness of hypodermic needles for drug transport across the skin [1,2,3]. Microneedles are micron-scale needles that puncture the stratum corneum, which is the exterior surface layer of skin. They are typically 50–250 µm thick and 150–1500 µm long [4], allowing for penetration of the skin barrier without causing bleeding or stimulation of deeply located pain receptors. The use of DMNs in the delivery of drugs, including biopharmaceuticals and vaccines, has recently been explored [5]. DMNs are a type of microneedle composed of biodegradable polymers that incorporate a target drug. Once inside the skin, interstitial fluid facilitates the dissolution or degradation of the polymers, as well as the subsequent release of the drug [5,6]. Microneedles employing a potent hormonal contraceptive drug also demonstrated sustained release, utilizing a patch as a backing support [7]. Drug-loaded microneedles can be easily self-administered with minimal training and significant tolerability [8,9,10], indicating their potential in the management of Alzheimer’s disease (AD).

**Figure 1:**
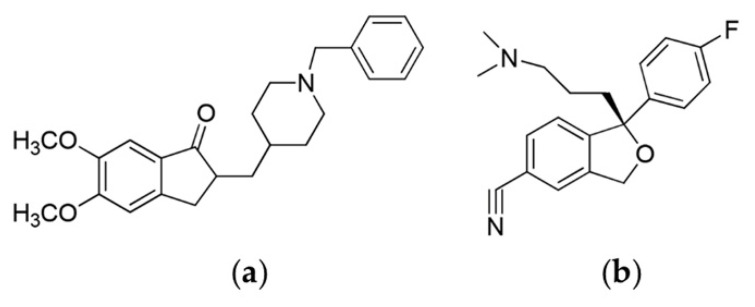
The chemical structures of (**a**) donepezil and (**b**) escitalopram (Internal Standard, IS).

AD involves deterioration of cholinergic neurons and is characterized by cognitive and behavioral dysfunctions that are highly prevalent among patients of old age [11,12,13,14]. Donepezil (Figure 1a; 2-[(1-benzylpiperidin-4-yl)-methyl]-5,6-dimethoxy-2,3-dihydro-1*H*-inden-1-one) is a widely prescribed acetylcholine esterase inhibitor used to treat all stages of AD [15]. However, the rates of adherence to pharmacotherapy among patients with AD ranges from 40–65%, with more than 90% of patients discontinuing the therapy after 2–3 years [16]. The poor drug adherence is typically due to frequent dosing, gastrointestinal adverse effects, dysphagia associated with dementia, and the absence of caregiver support [17,18]. The major reason for discontinuation was found to be the adverse effects that occurred more frequently at high dose [16,19,20]. Since plasma fluctuations of donepezil are large and frequent, the incidence of adverse effects increases with oral administration [21]. In terms of patient acceptability and adherence, the transdermal patch could be an alternative to oral administration, owing to reduced gastrointestinal adverse reactions and ease of self-administration [22,23,24].

Donepezil is a small lipophilic molecule with a molecular weight of 379.2, making it well suited for transdermal delivery [21,25]. However, sustained release forms of transdermal patches for prolonged dosing intervals are still rarely available commercially. Thus, we developed and evaluated a novel DMN patch for the sustained release of donepezil to address noncompliance in patients with AD. DMN patches consist of a hydrophilic and biodegradable polymer, sugar, and an incorporated drug. They are fabricated in an array of 100 DMNs with an overlying film made of the same drug–polymer complex and supported by a backplate of biocompatible resin. We evaluated the pharmacokinetic profile of donepezil after the application of the DMN patch compared with other routes of drug administration in rats. Additionally, we demonstrated the advantages of microneedle patches through an animal study by comparison with a needleless patch. The goal of this study was to see how well donepezil was absorbed from DMN patches, understand the newly developed formulation, and evaluate the effectiveness of DMN patches in vivo.

## 2. Materials and Methods

### 2.1. Materials

Donepezil hydrochloride (Figure 1a) was purchased from Tokyo Chemical Industry (Tokyo, Japan), and escitalopram oxalate (Figure 1b) was purchased from Sigma-Aldrich (Saint Louis, MO, USA). DMN patches were prepared in the Laboratory of Pharmaceutics, College of Pharmacy, Gyeongsang National University (Jinju, South Korea). Fisher Scientific (Hampton, NH, USA) provided HPLC-grade methanol, acetonitrile, and water (Seoul, South Korea). Methyl *tert*-butyl ether was purchased from Avantor Performance Materials (Center-Valley, PA, USA). Tegaderm™ film and Micropore™ surgical tape were purchased from 3M Healthcare (Saint Paul, MN, USA). 

### 2.2. Animals

Koatech supplied 8 week old male Sprague-Dawley (SD) rats weighing 280 ± 20 g (Pyeongtaek, South Korea). The rats were allowed to acclimatize at the Animal Center in Gyeongsang National University prior to the experiment. The Animal Care and Use Committee of the University (GNU-170705-R0030) approved all experimental procedures for the animal study.

### 2.3. Dissolving Microneedle Patch

The DMN patches used in this study were manufactured by casting polymer solution containing donepezil HCl into the polydimethylsiloxane (PDMS; Sylgard^®^ 184, Dow Corning, Midland, MI, USA) mold. In detail, the molds were replicated by pouring PDMS (with the ratio of 10:1.5 weight of elastomer to curing agent) into the 3D-printed master microneedles template, which was designed by using Rhinoceros computer-aided design software (Robert McNeel & Associates, Seattle, WA, USA) and fabricated with a digital light processing 3D printer (MiiCraft PLUS, RAYS Optics Inc., Hsinchu, Taiwan). The master microneedle template was printed with individual layer heights of 15 μm in the *z*-direction and UV-cured for 6 s on each layer. The fabricated template was subjected to mild sonication using ethanol to further remove resin residues and UV-cured in an oven for 1800 s. The PDMS mold was detached from the master template after curing at 80 °C for 2 h in the oven. A solution of the polymer containing the drug was poured into the PDMS mold. After drying overnight in a 45 °C oven, the photocurable biocompatible resin solution was poured on the top of the polymer layer in the mold and subsequently cured under UV radiation for 200 s. Donepezil HCl-loaded DMN patches were obtained through their detachment from the PDMS mold. Microneedles were formed using only PVP and drugs, but some brittleness occurred during the demolding process or after microneedle manufacturing, necessitating the use of a plasticizer. The formulations containing various plasticizers are shown in Table 1. The microneedles containing the plasticizers were prepared by the fabrication procedure described above. After that, a digital optical microscope was used to evaluate the appearance of the DMN patch with plasticizers (MSDM-150, NanoInside Inc. Ltd., Suwon, Korea). The final DMN patches were characterized using a scanning electron microscope (JSM-6380LV, JEOL Ltd., Tokyo, Japan). The DMN patches were placed on an adhesive black carbon tape attached to a metal stub and sputter-coated with gold for observation.

### 2.4. Microneedle Patch Mechanical Properties

The mechanical properties of the microneedle patches containing drugs were measured using a texture analyzer (CT3 10K, AMETEK Brookfield, Middleboro, MA, USA). To test the microneedle patches under compression, a single patch was mounted on a rigid stainless-steel platform positioned vertically (microneedles facing up), and the test station sensor probe approached the microneedles at a speed of 0.1 mm/s in the vertical direction. The sensor and microneedle points were initially separated by 1 cm. When the sensor initially touched the microneedle tips, displacement and force measurements began and continued until the sensor reached the load of 90 N.

### 2.5. Determination of Drug Content in the Donepezil-Loaded DMN Patch

Prior to application in rats, the donepezil contents in the microneedles and whole units were determined using an Agilent 1260 HPLC system (Agilent Technologies Inc., Waldbronn, Germany). Chromatographic separation was achieved using a CAPCELL PAK C18 column type MG II (4.6 × 150 mm, 5 μm, Shiseido Co., Tokyo, Japan) with a gradient consisting of mobile phase A (20 mM ammonium acetate pH 6) and mobile phase B (acetonitrile) at a flow rate of 1 mL/min. In 3 min, the mobile phase B was increased from 40% to 90%, held constant for 2 min, and then reverted to its original state in 0.5 min. UV detection at 271 nm was performed with an injection volume of 5 µL and a temperature of 30 °C during the analysis.

To determine the drug content in the DMN patches, five patches were used for each formulation. The microneedle part (after separated from the DMN patch) and the whole unit (microneedle + patch) were added to separate glass vials and dissolved in 50% methanol. The solutions were stirred, sonicated for 1 h, and transferred to a 10 mL volumetric flask. The vials were rinsed three times, and the rinsing solutions were added to the flask. Finally, the solution was diluted to the mark. Further serial dilutions were performed to prepare working samples for analysis.

### 2.6. Dissolution of Microneedles after Application of DMN Patches in Rats

Donepezil-loaded DMN patches were applied to shaved skin in SD rats. At predetermined application times, the DMN patches were removed and visualized under a digital microscope (MSDM-150, NanoInside Inc. Ltd., Suwon, Korea) to assess dissolution of the microneedle patches for each application time.

### 2.7. Skin Irritation Test following DMN Patch Application in Rats

SD rats were shaved in specific areas. Donepezil-loaded DMN patches were then applied to the shaved skin and removed 1 h or 1 week after application. The skin application sites were observed under a digital microscope (Dino-Lite 2.0, AnMo Electronics Corporation, Hsinchu, Taiwan) to assess the degree of skin irritation after application of DMN patches.

### 2.8. In Vitro Drug Release from DMN Patches

An in vitro drug release study using the artificial membrane was conducted to evaluate the formulation of the donepezil-loaded DMN patch. The formulations used in the in vitro drug release study were designed by fixing the donepezil content in the solid to 28.6% and varying the ratio of trehalose and PVP, as shown in Table 2. DMNs used for the in vitro drug release study were prepared by the fabrication procedure described above.

**Figure 2:**
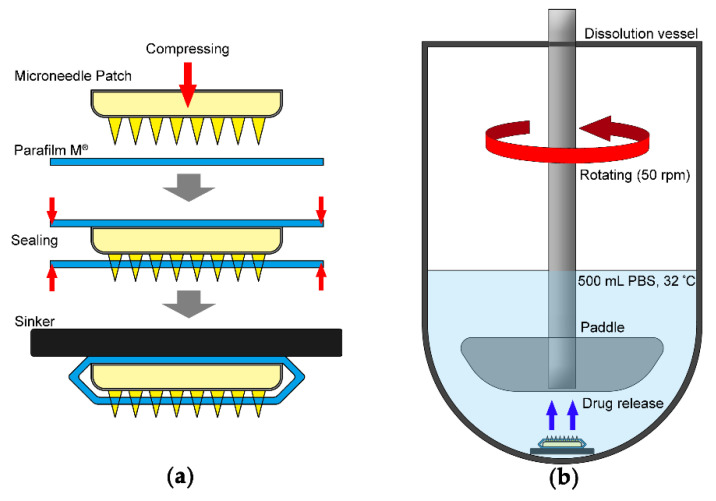
Schematic diagram of (**a**) DMN patch sample for the drug release and (**b**) test conditions.

Parafilm M^®^ (PF) was selected as an artificial membrane. The overall procedure of the in vitro drug release study of DMN was adopted by modifying a previously reported method [26] (Figure 2). Briefly, PF was punctured with a DMN patch using a texture analyzer (CT3 10K, AMETEK Brookfield, Middleboro, MA, USA). The DMN patch was placed on a single layer of PF on a sheet of PDMS-based skin phantom with an elastic modulus of ~1.8 MPa; then, a force was applied to the DMNs with a probe descending at a speed of 0.5 mm/s until it reached 50 N and ascending at the same speed after 30 s of hold time. After sealing the DMN patch with another layer of PF by heating, a metallic sinker was attached to prepare the sample (Figure 2a). The drug release test was performed using USP II dissolution apparatus (DT 126 lite, Erweka, Langen, Germany), with preset rotation of the stirring paddle to 50 rpm using 500 mL of phosphate-buffered saline (PBS, pH 7.4) as the dissolution medium at a constant temperature of 32 °C (Figure 2b).

Samples were collected at the predetermined time, and an equal volume of medium was replaced. The collected samples were diluted appropriately and analyzed at a wavelength of 271 nm using a UV/Vis plate reader (Victor Nivo^TM^, PerkinElmer Life & Analytical Sciences Ltd., Pontyclun, UK). According to the results of UV spectrum scanning at an interval of 1 nm in the range of 250 to 300 nm, it was confirmed that PVP and trehalose which constitute the DMN matrix did not interfere the analysis of released donepezil at a wavelength of 271 nm (data not shown).

### 2.9. Analysis of Donepezil in Rat Plasma

The levels of donepezil in rat plasma were measured using liquid chromatography–tandem mass spectrometry (LC–MS/MS), with escitalopram serving as an internal standard (IS). An accurately weighed reference standard was dissolved in methanol to prepare a stock solution of donepezil HCl (5 mg/mL as freebase). Serial dilutions of the stock solution in 50% methanol yielded working solutions of 2000, 1000, 500, 200, 100, 20, 10, 5, and 2 ng/mL of donepezil. Calibration standards were prepared by spiking 10 µL of the working solution in 90 µL of blank plasma. All calibration standards were transferred into microcentrifuge tubes in aliquots of 20 µL and stored at −80 °C. Then, 100 µL of acetonitrile containing IS (escitalopram; 1 ng/mL) was added to a 20 µL aliquot of plasma sample or calibration standard and vortexed during sample preparation. Afterward, 100 µL of carbonate buffer (pH 10) was added, and the mixture was vortexed again. By adding 1 mL of methyl *tert*-butyl ether, vortexing the solution, and keeping it at 4 °C for 10 min, liquid–liquid extraction was accomplished. It was then centrifuged for 10 min at 4 °C at 12,000× *g*. One milliliter of the supernatant was collected, transferred to a clean tube, and evaporated under a nitrogen stream at room temperature. The residue was then reconstituted in 50 µL of the mobile phase consisting of 0.1% formic acid in water and 0.1% formic acid in acetonitrile (75:25), respectively. Thereafter, the solution was centrifuged at 12,000× *g* for 1 min at 4 °C. A volume of 5 µL was then injected into the LC–MS/MS system for analysis.

Analysis was performed on an Agilent 1260 HPLC system (Agilent Technologies Inc., Waldbronn, Germany) with an Agilent 6460 triple-quadrupole mass spectrometer (Agilent Technologies Inc., Singapore) equipped with an electrospray ionization source. Using a C18 column packed with core–shell particles (Agilent Poroshell^®^ 120 EC-18, 3.0 × 50 mm, 2.7 µm, Agilent Technologies Inc., Santa Clara, CA, USA) and a gradient elution of mobile phase A (water with 0.1% formic acid) and mobile phase B (acetonitrile with 0.1% formic acid), chromatographic separation was established. The gradient program is listed in Table 3. For this gradient program, a flow rate of 0.3 mL/min was set, and the column oven temperature was kept at 40 °C. The overall duration of the run was set to 8 min.

Detection was performed by monitoring the precursor-to-product ion transition using multiple reaction monitoring (MRM) in positive mode. The MS/MS source parameters were set as follows: the drying gas temperature and drying gas flow were set at 300 °C and 13 L/min, respectively; the sheath gas temperature and sheath gas flow were set at 300 °C and 12 L/min, respectively; the capillary and nozzle voltages were set at 4500 and 0 V, respectively. The mass spectrometric parameters for donepezil and IS are listed in Table 4.

The method was validated in compliance with the bioanalytical method validation guidelines established by the Food and Drug Administration (FDA) [27]. The validation parameters include selectivity, sensitivity, calibration curve, recovery, matrix effect, precision, accuracy, and stability under different conditions of sample storage.

### 2.10. Pharmacokinetic Study

Prior to the experiment, the rats were acclimated for 7 days with unrestricted access to water and chow in a dedicated animal house in the university under standard temperature and humidity conditions. Before starting the experiment, the rats were shaved without damaging the integrity of the skin to allow for application of DMN patches to the skin. Afterward, surgery was performed on anesthetized rats. The carotid arteries of the rats were cannulated during surgery. The rats were then placed individually in the metabolic cage wearing harnesses to protect the cannulas during the experiments. The rats were given a recovery period of 24 h. Prior to each pharmacokinetic study, the rats fasted for 12 h but had access to water. Volumes of 0.6 mL of 20 IU heparinized saline were then injected into the lines after collecting blood samples to prevent blood clotting in the lines.

#### 2.10.1. Application of DMN Patch to the Rats

The DMN patches were applied to the backs of the rats by pinching the skin with firm thumb pressure. One DMN patch was applied to each rat (*n* = 5) at doses of 43 and 72 mg/kg for the 1 week and 1 h study groups, respectively. The DMN patches were secured by applying Tegaderm™ films and Micropore™ surgical tape over each patch. The DMN patches remained attached to the rat skin for the indicated times. Blood was collected after 0, 0.25, 0.5, 1, 6, 12, 24, 48, 72, 96, 120, 144, and 168 h in the 1 week application group and after 0, 0.25, 0.5, 1, 6, 12, 24, 48, and 72 h in the 1 h application study group. To obtain the plasma, 100 µL of blood was taken and centrifuged at 1000× *g* for 1 min. These specimens were kept at −80 °C until use after centrifugation.

#### 2.10.2. Subcutaneous Administration of Donepezil to the Rats

For the subcutaneous (SC) administration study of donepezil, five rats were cannulated as in the DMN patch application study. Before the experiment, sham hair removal was performed to compensate for effects due to hair removal in the other study groups. Donepezil HCl was administered subcutaneously to rats at doses of 1 mg/kg (as freebase). Sample collection was then performed 72 h post administration, as per the study protocol.

#### 2.10.3. Application of Transdermal Needleless Donepezil Patch to Rats

Donepezil needleless patches at doses of 72 mg/kg were applied to the backs of five rats. The rats were then subjected to the same protocol as the previous DMN patch application study.

#### 2.10.4. Oral Administration of Donepezil to Rats

Rats in the oral group were also subjected to sham hair removal. Oral doses of 3 mg/kg (as freebase) of donepezil HCl solutions in saline were given to five rats. A standard study protocol was followed, in which plasma samples were collected for 72 h.

### 2.11. Statistical Analysis

To compare the pharmacokinetic parameters of various study groups, we used one-way ANOVA. In all cases, *p* < 0.05 denoted statistical significance. Statistical analysis was performed using SPSS version 16.0 (IBM Corporation, Armonk, NY, USA).

## 3. Results

### 3.1. Dissolving Microneedle Patch

**Figure 3:**
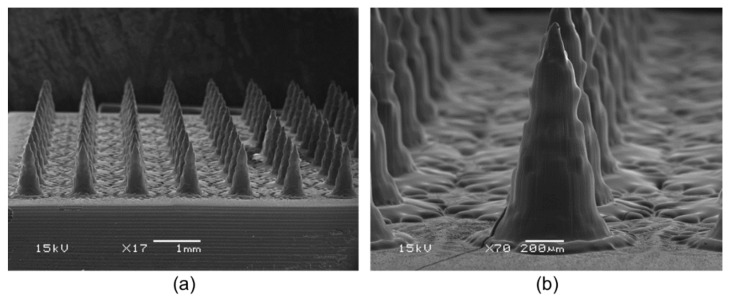
Typical SEM photomicrograph of donepezil-loaded DMN patch (acceleration voltage 15 kV). Arrangement of microneedles in (**a**) the patch with a scale bar of 1 mm and (**b**) a single needle with scale bar of 200 µm.

DMN patches were manufactured with various additives. The formulations containing maltose (S3) resulted in incomplete MN formation. The formulations containing sorbitol (S2), glycerin (S4), and propylene glycol (S5) were well separated from the PDMS mold without needle breakage, but the surface of the needles appeared to melt. Since only DMNs containing trehalose (S1) were completely separated from the mold and had an intact shape with suitable mechanical properties, trehalose was adopted as the additive to be used to fabricate the DMN patch. The microscopic characteristics of the DMN patches fabricated with the S1 formulation are shown in Figure 3. The needles were perpendicular to the base and had the shape of a conical tip on a beveled shaft. The needles had a height, base width, and tip radius of 1101 ± 17 µm, 634 ± 26 µm, and 10.6 ± 3.4 µm, respectively (mean ± standard deviation, *n* = 15). The interspacing between the microneedles was 1.13 mm wide. Each DMN patch had 100 microneedles per 1.21 cm^2^.

### 3.2. Dissolution of Microneedles after Application of DMN Patches

**Figure 4:**
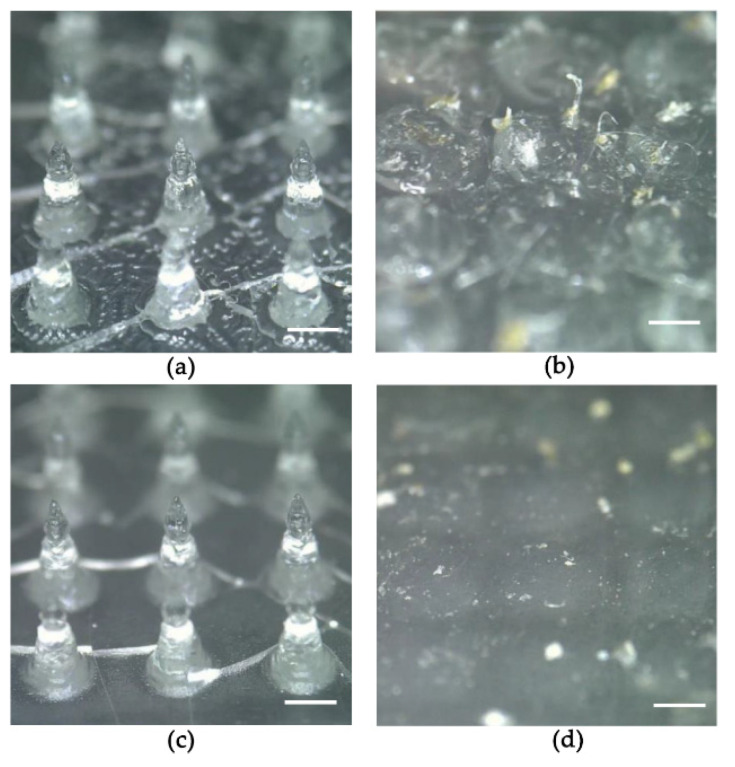
Dissolution of donepezil-loaded DMNs after application to rats. Images of microneedles (**a**) before and (**b**) after 1 h application, and (**c**) before and (**d**) after 1 week application. The scale bars represent 500 µm.

Figure 4 shows images of the microneedles before and after application of the DMN patches to the rats. Prior to application, the microneedles demonstrated visibly sharp pointed needles. In the 1 h application group, most needles disappeared, and the patches in the microneedle base were visible. In the 1 week application group, both the microneedles and patches were completely dissolved.

### 3.3. Skin Irritation Test following DMN Patch Application in Rats

**Figure 5:**
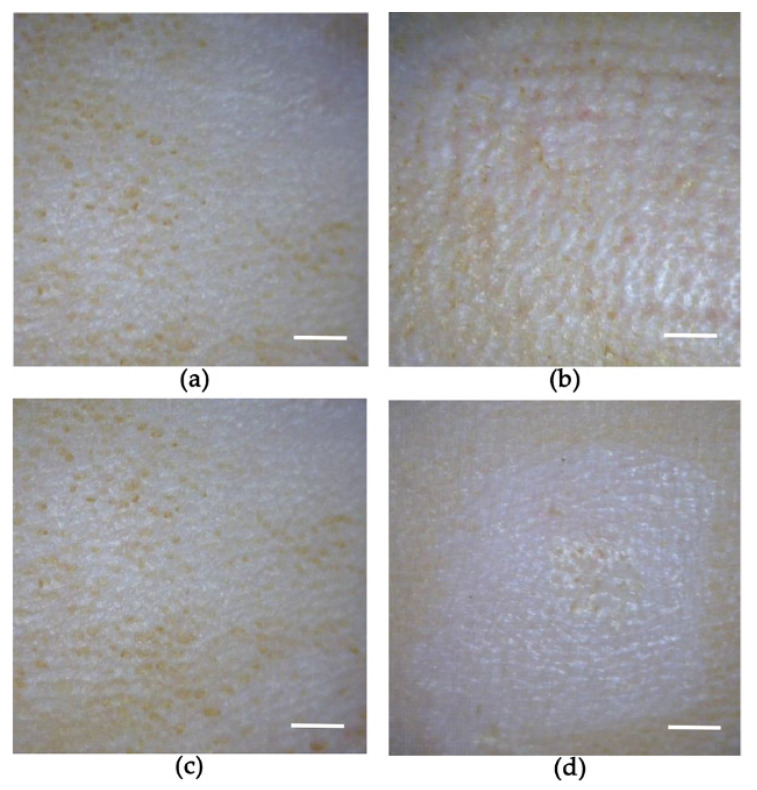
Absence of skin irritation following application of donepezil-loaded DMN patches: (**a**) before and (**b**) after 1 h application, and (**c**) before and (**d**) after 1 week application. The scale bars represent 2 mm.

The images shown in the Figure 5 compare the rat skin before and after the application of donepezil-loaded DMN patches. No skin irritation in the form of erythema or edema was observed after removal of the donepezil-loaded DMN patches upon completion of the study.

### 3.4. Microneedle Patch Mechanical Properties

**Figure 6:**
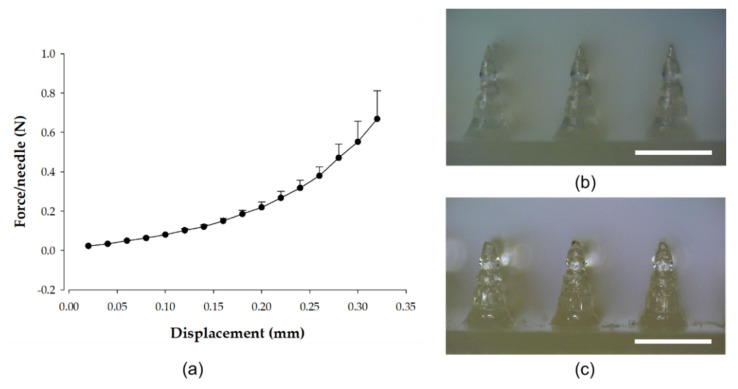
The mechanical behavior (*n* = 3) of the microneedle patches (**a**) and the shape of the microneedles (**b**) before and (**c**) after compression applied by a vertical force. The scale bars represent 1 mm.

As shown in the Figure 6a, the microneedle patches tolerated compressive forces of ≥0.15 N/needle, which is expected to enable skin puncture without breaking [28]. Following the application of stress (90 N/microneedles), the microneedles did not bend or break off, and their shape was maintained (Figure 6b,c).

### 3.5. Determination of Drug Content in Donepezil-Loaded DMN Patch

The average donepezil contents measured in the whole units and DMNs were 13.05 ± 0.34 mg and 2.26 ± 0.12 mg, respectively. The total contents of donepezil in the 1 h application DMN patch and transdermal needleless patch study groups were 21.74 ± 0.41 and 21.74 ± 0.34 mg per patch, respectively.

### 3.6. In Vitro Drug Release from the Dissolving Microneedle Patch

**Figure 7:**
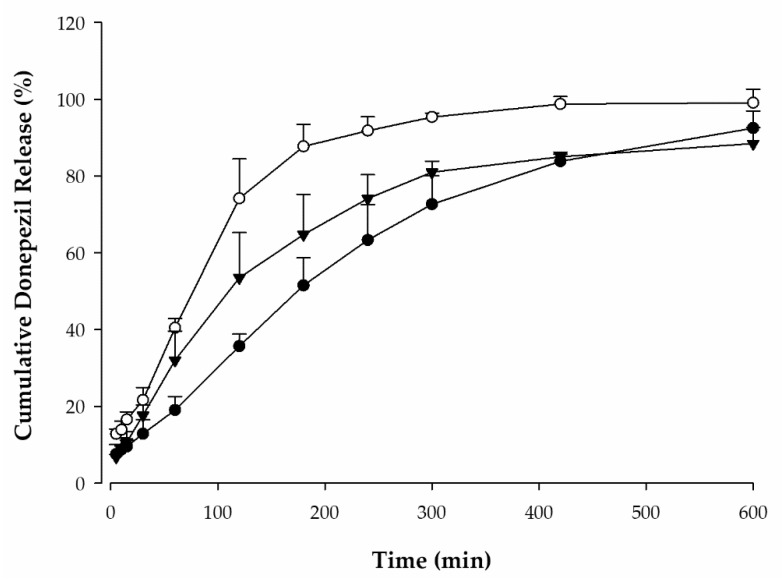
Cumulative donepezil release from donepezil-loaded DMN patches. F1 (●), F2 (○), F3 (▼). (mean ± standard deviation, *n* = 3).

The cumulative donepezil release of each formulation in pH 7.4 PBS is shown in Figure 7. The drug release of F1, F2, and F3 was almost completed at 600 min and was observed to be 92.5 ± 4.5%, 99.1 ± 3.6%, and 88.5 ± 4.2%, respectively. Compared with F1 without trehalose, F2 and F3 showed faster release rates in the initial stage. This means that trehalose in the drug matrix affects the release pattern. Additionally, the drug release rate of F2 was faster than that of F3 at all timepoints. Therefore, F2 was adopted as the DMN formulation for further pharmacokinetic studies in rats.

### 3.7. Analytical Method Validation

The analytical method of donepezil measurement in rat plasma was validated successfully according to the US FDA bioanalytical method validation guidelines provided as draft guidance for the industry [27]. Parameters including specificity, selectivity, linearity, precision, accuracy, recovery, matrix effect, and stability were evaluated in the method validation. The calibration curve was linear over the range of 0.2–200 ng/mL (*R^2^* > 0.996). The equation of the curve was *y* = 0.217317*x* + 0.010587 (*n* = 6). The accuracies (intra- and inter-batch) and stabilities (at various sample preparation conditions) were between 85% and 115% with precision below 11.0% and 13.6%, respectively. The recovery was greater than 80%, and the matrix effect was consistent across all QC levels. This validated analytical method was employed to quantify donepezil in rat plasma after application of DMN patches.

### 3.8. Pharmacokinetic Study

**Figure 8:**
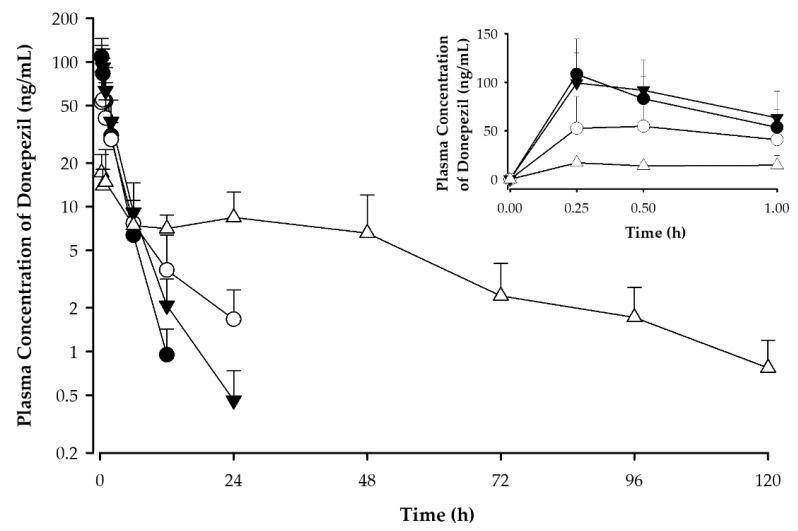
Plasma concentration–time profile of donepezil after 1 week application of DMN patches (△), 1 h application of DMN patches (▼), and subcutaneous (●) and oral (○) administration to rats (mean ± standard deviation, *n* = 5). Timepoints up to 1 h are shown in the insert graph.

The plasma concentration–time profiles and pharmacokinetic parameters of donepezil in rats after various routes of administration are shown in Figure 8 and Table 5, respectively. Donepezil was detectable from the first blood sampling timepoint (15 min) for all dosing routes. The 1 week application DMN patch study group demonstrated plasma concentrations of donepezil higher than the lower limit of quantification (LLOQ; 0.2 ng/mL) up to 120 h after application. Meanwhile, donepezil was detectable up to 12, 24, and 24 h after SC, oral, and 1 h DMN patch application, respectively. Furthermore, donepezil was rapidly absorbed in the rats, reaching peak concentrations (C_max_) within 15–60 min after all routes of administration. The median T_max_ for the DMN patch study group was 15 min, indicating that the DMN patches quickly dissolve. This makes the use of DMN patches favorable for bolus delivery. The AUC_0–∞_ value of the 1 week application DMN patch group was significantly higher (2.2 times) than that of the 1 h application DMN patch group. Donepezil may have been continuously released and absorbed from the patch after 1 h despite the disappearance of most needles within 1 h of application (Figure 4b). The 1 week application DMN patch study group demonstrated more continuous and sustained release of donepezil compared to the oral administration group, as evidenced by its longer half-life. The terminal half-life of donepezil administered via DMN patch was significantly longer (26.44 ± 6.36 h) than that administered via oral (6.01 ± 1.87 h) or SC (2.02 ± 0.39 h) routes.

The effectiveness of the DMN in enhancing permeation was confirmed by comparing the DMN patches with needleless donepezil patches. In the needleless donepezil patch group, donepezil was rarely detected in the rat plasma up to 72 h (data not shown). Among all the plasma samples, donepezil was detected in only few samples at values slightly above the LLOQ. The concentrations were not sufficient to calculate the pharmacokinetic parameters of the needleless donepezil patch. Thus, donepezil was rarely absorbed from patches in the absence of DMNs.

## 4. Discussion

Miyano et al. conceptualized DMNs in 2005 to allow for spontaneous dissolution in-to the skin [29]. However, previous studies have not focused on dissolving microneedle patches for sustained release [30,31], instead focusing on microneedles involving bolus drug delivery [32] and vaccines [33,34]. Our novel DMN patch is a combination of DMNs and additional drug layer, both made of biodegradable polymer, sugar, and donepezil HCl. A backplate of biocompatible resin supports this unit. We evaluated these novel DMN patches for sustained release, absorption pattern, and effectiveness in increasing the permeability of donepezil through the skin in rats.

For the in vitro permeation study, drugs were incorporated into a soluble polymeric matrix in DMN patches in which PVP was employed as a base material, and trehalose was used as a plasticizer for improved performance. The dissolution rate of F1 formulation, which contained no trehalose, was relatively slow during in vitro drug release study, because trehalose accelerates drug release from DMNs [35]. Since the mechanical properties of DMN are improved upon the incorporation of trehalose [36], it is reasonable to exclude F1 in terms of microneedle fabrication and skin application. Because of its high water absorption capacity, PVP promotes rapid DMN dissolution and can, thus, improve drug bioavailability [37]. PVP is also known as a solidifying polymer, and DMNs containing PVP have a high mechanical strength, which is essential for optimal insertion into the skin. As shown in the drug release pattern in Figure 7, the mixture of PVP and trehalose increases drug release, but higher trehalose content does not mean faster drug release. Therefore, DMNs fabricated with F2, which facilitates a faster drug release than F3, were expected to have a high bioavailability. Thus, this DMN formulation was adopted as the final formulation. Accelerating the drug release from DMNs is an essential strategy to improve drug bioavailability [36].

For the in vivo evaluation, we firstly applied DMN patches to the shaved skin of SD rats for 1 h or 1 week to evaluate microneedle disintegration and dissolution upon application. Upon inspection, most needles disappeared in both cases. These observations suggest that the DMN patches demonstrate good skin piercing ability and good attachment to the rat skin. The rapid degradation of the DMN inside the skin could have been induced by the interstitial fluid of the epidermis, according to a prior work by Mary-Carmel et al. [38]. Additionally, we observed no skin redness or swelling caused by the DMN patches as compared with the control. However, we observed microscopic punctures on the application site evident of DMN skin penetration.

Secondly, to demonstrate sustained release, we administered DMN patches with donepezil dose of 43 mg/kg for 1 week (*n* = 5) and compared them with an oral dose of 3 mg/kg (*n* = 5) to rats. Samples were collected for 7 and 3 days in the 1 week application and oral administration groups, respectively. LC–MS/MS analysis of their plasma samples showed that the 1 week application DMN patch group exhibited donepezil plasma availabilities above the LLOQ for 5 days (120 h; Figure 8) compared with the oral administration group (24 h). The 1 week DMN patch group also demonstrated sustained release and constant absorption of donepezil compared with the oral administration group. Additionally, the terminal half-life of donepezil was four times longer in the 1 week application DMN patch group than in the oral administration group (26.44 ± 6.36 h vs. 6.01 ± 1.87 h; Table 5). This allows for longer dosing intervals and addresses patient noncompliance due to frequent dosing. Moreover, Donnelly et al. found that some types of DMNs can also allow for deposition of controlled-release systems in the skin [39]. This study demonstrates the effectiveness of DMN in successful drug permeation via the transdermal route.

Thirdly, DMN patches with donepezil loading dose of 72 mg/kg were administered to the DMN group for 1 h to evaluate their absorption pattern. We already found that 1 h application allowed for the microneedles to dissolve (Figure 4). Plasma samples were also collected for 72 h. We found that plasma availability of donepezil (24 h) and terminal half-life (4.35 ± 0.33 h) were significantly shorter in the 1 h application group than in the 1 week application group. This suggests that the DMN patch attachment to skin was critical for prolonging terminal half-life and continuous absorption. Conversely, our results indicate that DMN patch depot formation may be less likely. Thus, we performed another pharmacokinetic study to rule out the effects of the skin on depot formation. Bolus doses of 1 mg/kg of donepezil (as freebase) were administered subcutaneously. The pharmacokinetic parameters showed that donepezil reached the maximum concentration (C_max_) in 15 min, with a terminal half-life of approximately 2 h. After 12 h, the plasma concentrations of donepezil dropped below the LLOQ. These results are consistent with those of the 1 h application DMN patch group, suggesting that donepezil placed in the hypodermis layer does not demonstrate any sustained release effects without a source or depot attached to the skin in the form of a DMN patch.

Lastly, the effectiveness of the DMN patch was established by comparing a patch formulation of donepezil HCl using the same components without microneedle arrays. In this study, donepezil HCl patches at loading doses of 72 mg/kg were applied to rats. Negligible amounts of donepezil were found in the plasma of these rats over 72 h. According to the findings of these pharmacokinetic studies, the DMNs dissolved in interstitial fluid, allowing donepezil from the patch layers to be absorbed into the microvasculature of dermis. Therefore, film layer dissolution may be the rate-limiting step of drug absorption in DMN patch application. Once inside the skin, we assume that the microneedle array is first dissolved in the interstitial fluid, creating mechanical aqueous channels. These channels allow contact between the donepezil HCl patch and aqueous phase, allowing donepezil to dissolve from the film after array dissolution. This results in continuous absorption of the drug in the dermal microcirculation, sustained release of the drug, and absorption into the systemic circulation.

In both studies performed with DMN patch applications, the T_max_ values were short, indicating that the microneedle arrays dissolve and release content rapidly, resulting in early C_max_ values. Furthermore, these results suggest that the upper film layers of the DMN patches function as reservoirs outside the skin. The DMN array also facilitated the absorption of donepezil from this layer through the formation of mechanical pores in the skin. Similar experiments with transdermal delivery of donepezil using hydrogel-forming microneedles and iontophoresis have been performed [38,40] as alternatives for improved treatment of Alzheimer’s disease. Contrary to the assumption that the of drug absorption rate is slower with the transdermal route, we found that our novel method achieved a sufficient T_max_ for donepezil delivery. Thus, initial bolus drug delivery may be followed by continuous drug absorption from these patches.

## 5. Conclusions

This study provides an evidence-based evaluation of the absorption pattern of DMN patches administered to rats. We showed that the microneedle array formulation with donepezil acted as a bolus drug delivery system with sustained and continuous absorption, as observed with an early T_max_ and sustained release from the donepezil patches atop the arrays. This dosage form can be further optimized to gain control over a range of doses and release patterns over different time intervals for various purposes. The benefits of transdermal products in Alzheimer’s disease are well established, with this study demonstrating the potential of DMN-based transdermal products on donepezil delivery in Alzheimer’s disease treatment.

## Figures and Tables

**Table 1:** The composition of donepezil HCl (DH)-loaded DMN patch containing various plasticizers (% *w*/*w*).

Compound	DH	PVP	TR	SR	ML	GL	PG	Needle Forming	Appearance
S1	27.3	54.5	18.2					Yes	Suitable
S2	27.3	54.5		18.2				Yes	Poor
S3	27.3	54.5			18.2			No	-
S4	27.3	54.5				18.2		Yes	Poor
S5	27.3	54.5					18.2	Yes	Poor

DH, donepezil hydrochloride; PVP, polyvinylpyrrolidone; TR, trehalose; SR, sorbitol; ML, maltose; GL, glycerin; PG, propylene glycol.

**Table 2:** The composition of the donepezil-loaded DMN patch used for the in vitro drug release study (% *w*/*w*).

Compound	F1	F2	F3
Donepezil HCl	28.6	28.6	28.6
PVP	71.4	53.6	42.9
Trehalose	-	17.9	28.6

**Table 3:** A gradient HPLC program consisting of 0.1% formic acid aqueous solution (**A**) and 0.1% formic acid in acetonitrile (**B**).

Time (min)	A (%)	B (%)
0	75	25
5	55	45
5.5	10	90
6	10	90
6.5	75	25
8	75	25

**Table 4:** Summary of the MS/MS parameters, and MRM transitions observed for each compound.

Compound	[M + H]^+^ (*m*/*z*)	MRM Transition	Fragmentor (V)	Collision Energy (V)
Donepezil	380.2	380.2 → 91.1	157	30
Escitalopram	325.2	325.2 → 109.1	80	25

**Table 5:** Pharmacokinetic (PK) parameters of donepezil after subcutaneous (SC), oral, and DMN patch application (1 h and 1 week) to rats (mean ± standard deviation, *n* = 5).

PK Parameters	SC 1 mg/kg	Oral 3 mg/kg	DMN Patch (1 h) 72 mg/kg	DMN Patch (1 Week) 43 mg/kg
AUC_0–∞_ (ng·h/mL)	213.64 ± 40.98	235.15 ± 79.17	273.64 ± 99.74	603.44 ± 320.39 ^2^
C_max_ (ng/mL)	108.32 ± 36.28	61.76 ± 27.50	100.46 ± 29.65	18.87 ± 8.36 ^3^
T_max_ (h) ^1^	0.25 (0.00)	0.5 (0.25–1)	0.25 (0.25–0.50)	0.25 (0.25–1)
t_1/2_ (h)	2.02 ± 0.39	6.01 ± 1.87	4.35 ± 0.33	26.44 ± 6.36 ^2^

^1^ Median (range). ^2^ DMN patch 1 week group is significantly different (*p* < 0.05) from SC, oral, and DMN patch 1 h group. ^3^ DMN patch 1 week group is significantly different (*p* < 0.05) from SC and DMN patch 1 h group. AUC _0–∞,_ total area under the plasma concentration–time curve from time zero to infinity; C_max_, maximum plasma concentration; T_max,_ time to reach C_max_; t_1/2_, half-life.
